# Supplementary material for: Enhanced Eicosapentaenoic Acid Production via Synthetic Biological Strategy in Nannochloropsis oceanica
Source: Mar Drugs. 2024 Dec 19;22(12):570. doi: 10.3390/md22120570 (PMC11676929; doi:10.3390/md22120570)
Supplement: Supplementary file 1 [file marinedrugs-22-00570-s001.zip › Supplementary Files/Supplemental Dataset 2.pdf]

>NO08G03500 promoter
CGGTGGGTGTTGATTTGCGTTGCCCAGACGCGTGCACTTGTCGCACCCAGGAGCCACCCAGCG
CGACTCAAGCAGGACTTTCACGTGTTGCGTGTTATCATGCAAGATGTGGGGCATGGTTCAGATC
AAGGCATCGCATTACAAAAAGGGCTTTGCACGAAGGACTGACTCCTCGTCGCACCATCATCAAA
AGTCACGTCCGTGCCGAGCCGTCGGCAGGTGTGCAGGTAAAACCTCACTCTTAAATGATAACCC
ACTACCTCACAGGATCTCTGCATGCCGCTCGTGGTGGCTGACTGAGATCTATGCTCATCTATCAG
TTCAGTCGAAGGTACCAGGATTGCTAGCTAGGGCGCTCCAGGAGACGCGCCTGCTAGCTCATC
CCCATTCTTTACTCGTACACAAAAGCTTATACTGGAGGATGGCAGGGTAGACTAGAAAGCCCCC
GAACTCATATGGTAGGGACAACGTTGGTAGGAAAAATGTTGCATTATTACAAATACATGTCAAG
AAATGTAAACCAAGCTAAATGCGTTCATCAAAACATGTGGAAACTCCCGCACGGCAAGTGGTTC
TTCTTTGCTGTGGTCTGAGGCAAAACAGGACCCTTGCCATCCCTCCCTCCCTCAATCCAACCTCC
ACGATCTCAACCCCAGCGTAAGTCCAAGGACAGGAAAAGTCGTGCTTCCTCATTACTTCTCTTTT
CTCTGGCTTCTCCTCTTAAGGGACGGTCGTTTCGATTGTATTATCTGTGGTTACTCTTGCGACTGC
CTACCTAGCTTCTTTCATTTGGGGACCTGTCTGAGAGAGGACACACACACACACAACCGAACAA
TCACATAGTCAAACACAGACAAATAATAATTCATGCCTCATCCCTCTCTCGCTTTGGATCTTTTTC
TTTTAATACATCAAGATGGAACAAGAACATGTGTGGTAGGTGTGTGATGTGAGACAGCGCATGG
ATGCCTGTGCCTTCTCTCCTATGAAAAAAAAACGT

>NO08G03500 terminator
CTTTGTGTCTAAAGATAAAAGAATACTGTGGGGTTGTGTTTTGTGTTAGCGTGTAGGAGGAGGGT
TAGGACAGTGACCATGTGGAAGAAAGCATGCAGCTCTTCTTGGTTCTGCTTCTGCTTGCAGGAT
CAAGATTTCACCACGGTGCCGAGTTGCAGACAAGATCCATTGTTGTACGGAGACGCGAAAGCTT
CACCATCTGCCTGCACGTTTGTTAGAGTCTGTGCCGTAGATAGGTGCGAAGATGAAAGGCACAG
GGTAAGCAGATCAAGGCAAGTTGAATGCCTTGGATTTGTGTCTGTTGGGTTTGCCTTCCCATGCC
CACTTGTGGTCACGCGCGCGCCTCCACCGATTTGTTCGAGCAAGTGAAAACCACACTGGAGTCC
AAAGACACACTTCTAGAGACTAGACGAATAAATATCGGCCTCCCCAGGTACCAAGGACACACG
CTGGAATGGCTACAGGAATAGATTTCGACTGCCCGAGCTCAACTGCTTCTTCTCGCCAGCCGCA
GCCCAAGCCCGCCAATTTTTGCGTAATTTGGGGAATTGTGACCCCAAATTACAGTAACTCCTATG
TCCAAGACCCGTTCGGCCCATGCTTTTGTTCGTCTCGTCCCTATTTTTGCCCTTGCGTGGTTTTGG
ATGACGCCGCCCTAAATGTGCCCGGCTTCAATTTTTATGTTTTCTGGTCCCATACCACCACACAG
TCCATGTCCCTCGCCGCCGATGGCGCGTCAGACGAGTTGTTTTTCCTCCTTTGGGCCGAGAACG
AGTGGCCCTCCTCCCGGCCGCGGCAAGAACGACACCATCACGCGCAGCATGCACCAGCATCG
ATACCCTGGTATTTACCTCATACTGTTGTGTACAAGGCATCGAGCCCGTGTGCTTGGTATTTTAC
CTCGGCCAAGACCAGCAATATCTTGAAAAAGAGCAAGCACAACGTGCTCAGTCGGCGCATCGA
GGAGGAGTTTACCAAGCAAACCCTCGCAGGCGACATT

>NO03G03480 promoter
GATAGGAACTACGACTGCGACGGGCATAGGTGTCCTGGTTGCTCAGTGCATGATCCTTGGCCCA
CATAACGCGAAAAAGTAAGGCTTATGGGCCCATGTCGACCGTTAGTAATGGTCGTATCGGTCGA
CGTCGACACCCGCATTGGTCCTCGAGTGGCGAGTAGTGCTGCAGCCCTTTTCGTGAATTGCCGG
CGCTCGGATTACTACGACCTGGACCCAACTGAATTGTCTACGACTTAAGATTGCAAACTAATTCC
ATTTTCCAAGCCATGTAGTCCGTGCCTCCGGGACAAGGGGTCATTTTATGGATAATGAGCCATT
GTACCACTGGCAGCAGTCCCCATTCGCTGCCAGTTTGAGCTTTGATCTTCGCAGGAGGCCAACT
GCCAGTCTCTTTCTGCTTCTGTTCCTATACTAATAATATATATATTCCCCCCATTAGCAATCCTCTC

ATTTCCCTGCTTCTTCATGGAGGAAATGTACTTTCATTTGTCTGCCCAATGAAGAAGGCTTCTTCG
CTTGGTGTAGGCTTCCCACCCCTTCAATCCTTCCACCACAGCTCTCAATCCTTTCTAAGGAACAG
AGCTCCTCACCACCCTGAATCGAGCATTTGGGATCTTTCTACCATGGGACGTTCTTTCCCGGCTA
GCCCGGCTCCGCCTCTCGTGTTCTCACACAGTGCACACAGTTCGAAAATCATGACAGGTGTGTG
TGCAGGTCCACGGGGCTTCGTTAGCTGCTTGCTGCACGTTGGCCTCCCCACGCACCTCCCCCGT
CCGCGCGCGTGCCATTTCTTGTTCGGGCATGCAACTTTGCCATGATCGCAACTGGACTCAGTAA
TCCAGCACCGGGTCATGGAATTTAAGTAGAGGCAGCCACAGCCCTACATATCCATCCACGCGC
ACACATTCCCAGCAACCCACACTGACCATCTCCCCAAAAAAATCCCGCAAACCCACAGGTCCCT
CCTCCCAACAACTCTCCACATCAACCATATACT

>NO03G03480 terminator
AGGATGAGTCGAGAGCCCTAGCCAACAGCGAAAACCGAAACAGCAGAACAAGGAGCCACAG
GATGAAGAAAAGCAGGAGCAGGAGCAGCAGCAACAATTACGGTGCAAACCCTCGCCACTGGA
TGGCTGGCGTGTGACTTTTTGTGTGTAGAAGAAAATAAGAAATACGGTCCCCGCATGCAGTGTA
GTCAGATGATAGAGTTAACGAAATGCTTGCTTTGTATCGACAACCAGCCTCCTTTCTTCTGTCTCT
ATGTCATCGTAGAAAACTACAGATGTTGGCACCTCATGAAAATTCAATTACACTTGTTGTTGGAC
TGTCATATTCTGCGAATCCTTCATGAGCGTGAGTTTTCCCCCTCTATGTGGCAAAAAAGCACTCG
CACTGAAAGAGCTGCTGATGTATCACACAAGTCATCTAACAATATCTAACACACTCAGATTACTA
TCCATATCCATGGCCGATATGCCGCCAATGCAAGTACCTGCACCTCCCTTGTCTCCTTCCCCCTA
CATCACGAATCTCGCCAGCGCTCTTACCACTGCCCTGCCCCCTGTAACTTCCAAACAACTGAAA
ACTGACTGTTCACACACATCGCCTGTAGGACGGTTCGCGACACCAATACTCGCGGCCGCCGCG
CGAACAGTACCCCGACTGGCGTTTGCCAAAGCAAACAATTGAAAAGTCTCCATTTCCATGCTTA
ACAACGAAGGATGCTGGGTCCGCAGTGTGTCGAGCACGTGCATGTTCCGGTCGTCGAACAGGG
TGTCTTCTCGGCCTTGGGAAGAGTAGAAGGAGCAGGCAGAGGCGTTCATTCCTTGTGTAACTCG
GGCAGGACCTAGCTCGGCGATGAGTTCAGAAACCAAGGCATGGGATAGTGCTCGATCAGAGG
GTTGAGGTGCAAATACCAGATATGGTTGGTGAGGTGTGTGAACCGTATCGTTGACAGCGTCTGT
TGGTACCGTTTTTGTCGTAGTGGTGGAGGTGGTGGGAGGCAAG

>NO22G01450 promoter
TCTTTGCTGCACTGCCGGCGCTGCACCACCTCGCCCTGCCAGCCACCGGCCAGGGTCGTGGCA
TCGATGGGCATGTACGCGCCTCACGCACGGCCCGCGCCGGCATTTTGTTCATAGGACTTTGATA
AACACCTCTGGCTCATTCTCCCTCTCCTCCTCACCCATCCACCACGAGCACAGCACCCCCACCC
CTCAACCCTCACTTCGTAACAATGGGAAAGGAGAAGACCCACGTGAACCTGGTCGTGATTGGC
CACGTCGATGCCGGCAAGTCTACCACCACGGGCCACCTCATCTATAAGTGCGGCGGCATCGAC
AAGCGTGTGATCGAGAAGTTCGAGAAAGAGGCGGCCGAGATGGGCAAGTCTTCTTTCAAGTAC
GCGTGGGTGCTGGACAAGCTCAAGGCTGAGCGTGAGCGTGGTATCACCATTGATATCGCCCTCT
GGAAGTTCGAGTCTCCCAAGTACTACTTCACCATTATCGACGCCCCCGGCCACCGTGATTTCAT
CAAGAACATGATTACCGGTACCTCCCAGGCTGATGTTGCCATCCTCATCGTCGCCTCCGGGACG
GGAGAGTTCGAGGCGGGCATTGGCAAGGATGGGCAGACCCGCGAGCACGCCCTGTTGGCGTT
TACCTTGGGTGTGAAGCAGATGATTGTGTGCGTCAACAAAATGGACGACTCGTCTGTCAACTAC
GGCGAGGCCCGCTTCAATGAGATCAAGGAGGAGGTGTCCAACTATCTCAAAAAGGTCGGATAC
AAGCCCGCCAAGGTCCCCTTCATTCCCATCTCCGGCTGGAATGGCGACAACATGATCGAGAAG
TCTACCAACATGGCCTGGTACAAGGGCCCCACTCTCCTGGAGGCCCTTGACAACATCAACCCCC
CAAGCGCCCCACGGACAAGCCCCTCCGCCTGCCCCTCCAGGACGTGTATAAGATCGGCGGCAT

TGGAACGGTCCCCGTCGGCCGTGTGGAGACCGGTGTCCTGAAGCCTGGC

>NO22G01450 terminator
ACGAAGCAGCAGCAGCAATAGCAGCGGCAGCAGTAGTGATTGGCAGTGGTGGTTTATTTTAGG
CGGTGATAAAAGGATTTGGGAGGATAGAGAGGACAGAGACCAGTGGGATATGGGAGAAGGAA
ATGCCGGAGGGACACAGGGTAGGGACGGGCTTCGCGGGGGGAATGCGTTTCCGGCTTTTGCCC
TACATCGACCACCGCCTTCTTAAATGCCTGCTGGTGGGTCAGGGTAATGGTGGTTTAAAACAGC
CAGGGCGCTGATGGAGGCAAAAAGAATGAACCACTCAGTTTAATCAAATATGTTCTAGGAATGA
TTTTTCGTTGGACAAGAACATGCGTCCGGCTTTGAATTTGAACACGGGAGCGCACGCACTCAGG
AACAAAGAAGACAAAGCTAGTGAAGCCGAGTCGTGAGGATGGGTTTGGGGCAGAGGGTTCAG
GAGGAGACTAGGAAAGGAGGGTGCTCGAAAGGGCCGATCCAGTGCAAGCAGATGAGAAAGAC
ATAACACATTCCTTCGTCGCAAAGGAGATAGGGCACGAAGGAGGGCAAACTAAATCCGCTCAC
TAAGTTGTGTTGGTCACAAGATTCTGTTGCTCTGATTGATGGTTGCAGTATCTCATCGTCCTCGTA
GTTTTTCGTAAAGCATAGCTTTTAAGGTCCGTGAGGCAGGTTTGTGAGAATTAAAAGCCTGCTGG
CCTGGGCGAAAAGATGTCTCGATGTCCAAAAAATTAGACGTTGCCACATTTCCTTGTTGGCTCAC
TATATCCTTTAAAATGCAGTTGGACCTTATCGCATGCATGTTTTACGTGCACAAAGAAGCTCAAG
GCCTCTGAAGACCCAGGTAGATATTGAAGAAGGGAGAAATCAAAGCGTACGAAGGCGAACGG
CGCGACGGAAGGTTTCTTCGTCTCGGGCCGTCATAGAAATGTCACCTGTAAGAGAGGCAAGGTT
GGATGTCGACAAGACTGCTTGGAGAGGTGGTGTATTCAGACTTAGA

>NO12G02410 (α-tubulin) promoter
GGAAAGGGTGAGGCGTGCACAAAGGCTGCGGAGTCTGGCGTGGCGGCTACCATGGTGGCAAA
GAGCAGGAATGGGGTCCAAAATAAGGTGAATGCGTTATTTGATGTTGCTGTATGCATTGTACTG
GATGAAGGGCGGCCGAGGTAAGCTACGATACTTCAGGTCGGGGCCGCAAAAGGTTTAATTATA
AGGATGGTGCTGTGAACAATTCTGTGGGTTGGGTTCGGTAGGTGGTGGGATTTCAATTCAGAGA
ATTAAAAGAAATCATTTTTTGCGGGGTGTTATGGGACAGCGCGTGTGCACTTAAGAGGCTGCGC
TCGCTTGCGGCACGCAGAGACGGCACACATTCGCGAGAGGCAGAACTCCTACCGGCGCCCTCT
TATTCATCAGGTATTTTTTGTCAAGGGGTGTGCAGGATTTGAGGATAGACAGCACAATGCCTTGT
TGTCCCTAGCAAAATCCAAAAACAAAACACGACACAAGAGACCGCACTGTTTCCCCGCTCCCA
GATCTCATGAGGCGAGGCACGTGCATGGGAGTATGAGAGTGTGCCCGGCCGTGAGGTCACGA
GCGATTTGGCGAACTGCTGACGGCTTATGCAACGCTTTTGGTTTCGTTCATTCGTTGCTCCCCCC
TCCTCTTAAGGGACTGGACGATTCGTCAAGCCATTTTTGTGCTTTTTGTTGGTACACTCCAGGATT
ACCCGTCCTCTCCTCCCGGACAGTTACCTCACGGCTTGCCACCTGAAGCATGGATGAGGCCTGG
GTGCAGGCGCAAGCTTGTGCTTTTTTTGAATAACAAGTCTCGCACCCCGCCGAAAACCGGGGTT
CCATGTCTTCCCTCCACGTTTTCGGTGCGCTTTTCCCCCCTCTTTTGCACACCCACCACCTCGCAG
ACCGACTCCCTCGTCCGTACCAGGGACGCATAGGCCATTGTTATGCGTGCGGACAGGCTGAGG
TCTGCGCAAAGGCGTTGGTGACGACTACAGCTTCTTGCGTGT

>NO12G02410 (α-tubulin) terminator
GGCCGCCGACATCTGGTGTGTGTGGGATCAGGAGGAGGGAGTGAAGAGGAGAAGGGATCTGG
TTTCAGAGATCCCCACTTCTGCCGTCGTCTTTCGGCCTTCCTTCCTTTTAGGTGTCATGCCTTAGG
TCCTTCAAGTCCTCACCTGTCGTCGTCATGTGTGTGTGTGCCCGTCATACAAGTCACTCGATCCA
ATTCACGCATCGGTTCAATCAAAATAAGACTAGACCCCGAGGGAAGAAGGGCAGAAGGAAATC
GAAGGGGTGGGATGTGTGTGAGAGAGGGAAGGAGAAATGAAAGAAGTGAACAATGTCATGGT

AGCCAGTAAGGAGAGAGTAGAAGCGAAGAAAGCAAAAGCACTGTTGTGAAGAAACGAAATGG
AAGATGGTCATCGCTCCTGGCTCTACTTGTGGTTTTTCTATCTTTAATTTCAGGCGTCCTGGTCTC
GTTACATCAGCTCCCTTATCTCATTGGTTTATCCCCTACTCTACTGCTGCTTCTTCCTTCCATCCGT
GACTGTATAACAACGAATTGTAGTACCGCAGATAGACAATAGAAAAATGCCAAAAAAGGCATC
ATTGATTTGCTCCTCCCCATTAAGTCACTGTACGCCACCATCGCCACTACCCTCCCTTCCACCTA
GCCTCCCGCCTTCCTACTGATCATTCGCCGGCACTGGCTTCAACTCCAAGTCGATCCCCAAAAA
CGAGCTCAAGCTGTCACTCGCCTCCTTCAACTCTTTAAAATCAATCGCCTTCTCCGAGATGATTG
TCCCATCCATCAAAAATAGGACCGTGCCCCCGAATGGCGCAATTTTCGTCTCGGTATGTAGCTTT
TCCACGCGCAAGCCCCGCGCAAAAAACAACTCAGTGACTTTGTAAACCAAGCCAGGATGGTTC
ACACCCTCAACCGAAAAAATCGCCTTCCACACAGGCTTGGGAACGATCTGATTAGCTCTTTCGG
CAGTCACCGGGTGGGTAATGACAGACATGCCGCCGACT
