# Supplementary material for: Enhanced Eicosapentaenoic Acid Production via Synthetic Biological Strategy in Nannochloropsis oceanica
Source: Mar Drugs. 2024 Dec 19;22(12):570. doi: 10.3390/md22120570 (PMC11676929; doi:10.3390/md22120570)
Supplement: Supplementary file 1 [file marinedrugs-22-00570-s001.zip › Supplementary Files/Supplemental Table 1.pdf]

Table S1. Nucleotide sequences of the primers used in this study.

	Forward primer (5' to 3')	Reverse primer (5' to 3')
**Gene cloning**		
NoFAD1	AAGCTTACATAATGCAGCTTCCTTCCTTTCTGACAC	GAATTCTTATGAAGAAGCGACGTTGGCG
NoFAD2	AAGCTTACATAATGGCCATCGACAAGGCTG	GAATTCTTAATGTAGCTTCTTCTCCCTCACC
NoFAE1	AAGCTTACATAATGTCATACGAAACCTCCTTCCCC	GAATTCTTAGACCGTCTTGGCCTTGGG
NoFAE2	AAGCTTACATAATGTCACCTGCTGCTCAACACAAG	GAATTCTTATTCTTTTTGACCCGAAACTCG
NoDGAT2K	AAGCTTACATAATGTTGCTGGCGTCGTCTCGGC	GAATTCTCAGACGATGCGAAGCGTCTTGTGC
**Construction of overexpression vectors**		
pXY510 promoter	GTTAGCAGCCGGATGGTACCCGGTGGGTGTTGATTTGCG	TTTTAAATTGGTACCGGTACCAGGAGAGAAGGCACAGGC
pXY510 terminator	CTACTACATCTGTTTGGATCCCCTTCATCTTTGTGTCTAAA	ACACACAAAGCACCTGGATCCAATTTGGGGTCACAATTCCCC
pXY511 promoter	GTTAGCAGCCGGATGGTACCTACACGAGGATAGGAACTACGACTG	TTTTAAATTGGTACCGGTACCAGTATGGTTGATGTGGAGAG
pXY511 terminator	CTACTACATCTGTTTGGATCCAGGATGAGTCGAGAGCCCTAGCCAA	ACACACAAAGCACCTGGATCCTTGCCTCCCACCACCTCCACCA
pXY512 promoter	GTTAGCAGCCGGATGGTACCCTTCTTTGCTGCACTGCCGGCG	TTTTAAATTGGTACCGGTACCGGACACCGGTCTCCACACGG
pXY512 terminator	CTACTACATCTGTTTGGATCCACGAAGCAGCAGCAGCAATAGCAG	ACACACAAAGCACCTGGATCCTATCTCCTTTGCGACGAAGGAAT
pXY513	TGGAGACCGGTGTCCCTCGAGATGTTGCTGGCGTCGTCTCGGC	CTGCTGCTGCTTCGTCTCGAGTCAGACGATGCGAAGCGTCTTGTGC
pXY514	TTCTCTCCTGGTACCGAATTCATGCAGCTTCCTTCCTTTCTGACAC	AGATGAAGGGGATCCGAATTCTTATGAAGAAGCGACGTTGGCG
pXY515	TTCTCTCCTGGTACCGAATTCATGGCCATCGACAAGGCTG	TTCTCTCCTGGTACCGAATTCTTAATGTAGCTTCTTCTCCCTCACC
pXY516	CACATCAACCATACTAAGCTTATGTCATACGAAACCTCCTTCCCC	CTCTCGACTCATCCTAAGCTTTTAGACCGTCTTGGCCTTGGG
pXY517	CACATCAACCATACTAAGCTTATGTCACCTGCTGCTCAACACAAG	CTCTCGACTCATCCTAAGCTTTTAGACCGTCTTGGCCTTGGG
pXY518	GGATCCAGGTGCTTTCTGCAGTACACGAGGATAGGAACTACGACTG	ACACACAAAGCACCTCTGCAGTTGCCTCCCACCACCTCCACCA
pXY519	GGATCCAGGTGCTTTCTGCAGCTTCTTTGCTGCACTGCCGGCG	ACACACAAAGCACCTCTGCAGTATCTCCTTTGCGACGAAGGAAT
pXY520	AGTGCAGCAAAGAAGCTGCAGCTTCTTTGCTGCACTGCCGGCG	ACACACAAAGCACCTCTGCAGTATCTCCTTTGCGACGAAGGAAT
pXY521	AGTGCAGCAAAGAAGCTGCAGCTTCTTTGCTGCACTGCCGGCG	ACACACAAAGCACCTCTGCAGTATCTCCTTTGCGACGAAGGAAT
**Preparation of overexpression cassette**		

-	TCTCGTAAACCCTGTCCCACTC	AGGGTAGTGGCGATGGTG
**PCR identification of overexpression vectors and lines**		
MX513	AAGGATGGGCAGACCCGCGAGCAC	GACATAGATGATGCGTCGCTTCCGT
MX514	GGGTCAAATCCAACATCACCGGCAA	CAGGGTCATATTCCTTACCCGAGAC
MX515	GGGTCAAATCCAACATCACCGGCAA	GGGATGGGTAGAGATGAAGGGGGCG
MX516	CCCCCGTCCGCGCGCGTGCCATTTC	GGCCCGCTCCAAGATGGGAAAATGC
MX517	CCCCCGTCCGCGCGCGTGCCATTTC	GTAAAGGGCAGTGGCCAGGAGAGGG
MX518	CACCATCTGCCTGCACGTTTG	AAAGAACGTCCCATGGTAGAAAGAT
MX519	CACCATCTGCCTGCACGTTTG	GTGCTCGCGGGTCTGCCCATCCTT
MX520	GAAAAGTCTCCATTTCCATGC	GTGCTCGCGGGTCTGCCCATCCTT
MX521	CACCATCTGCCTGCACGTTTG	GTGCTCGCGGGTCTGCCCATCCTT
**Construction of *NoTGL1* CRISPR vector**		
-	CGAAACGTCCTGATGAGTCCGTGAGGACGAAACGA<br>GTAAGCTCGTCGACGTTTGACGCTATCCGAGG	AAACCTCGGATAGCGTCAAACGTCGACGAGCTTACTC<br>GTTTCGTCCTCACGGACTCATCAGGACGTT
**Sanger sequencing of *NoTGL1* CRISPR lines**		
-	AGCGAAAAAGATGGTTAGGAGGAAG	GCACTTCGCAACCCCAAACAT
**RT-PCR or RT-qPCR**		
NoFAD1	GCCTACGATTTGACGGACTGGG	GGCTTCCGATGTGTCCATTACG
NoFAD2	CTCTCGGTGGGGTTGCTCTCG	GTTGGTGAAAGAGTGGTGGGCG
NoFAE1	CTGACGCTCTCCCTCTTCTCC	GGAGGACGGTGATGTGGTGG
NoFAE2	CGCCTCCTTTATTTTCCTCTATCGC	CCCGCAAAACTTATGAGTGACAACG
NoDGAT2K	CTGCGGTCTACGCCGTCAT	GACAGATACTCACTGAACAGCCCTC
NO08G03500	GTTTCTCCAACAATCCCCCCCGC	CGGGCAAGTGGTAGATGTTGGGGAA
NO03G03480	TCGAGGCGCCTAAGGCATTGGAC	GATCTCGGCTTCCCTGAACCACTTG
NO22G01450	GAACGTGTCGGTCAAGGAGATCCG	CCGGTACGTCGGTCAATCTTTTCCG

NO02G01740	CTTTTATGCCGTCTGCCCCTC	CAAACGGGAGCGGTACAGGG
NO12G02640	GGACGGCTCCTTGGTGGGTG	GAGAACTCGGGGGCGAACTTG
NO16G01210	CGGCTCCATCCGTGTCAAG	CATTGGCAACGCAGTAGACAG
NO11G00800	CCCCCTTGGCTTCCCGTGCT	CTCAAAAGCCTTCTGCCACTCGTCC
NO22G01440	GCGGCGGCATCGACAAGC	GTGATACCACGCTCACGCTCAGC
NO09G00450	CGCTCTCCTCACTGTCTCTACCC	CCAAGGCCGGTCACCAGG
NO27G00910	CTGCCTTTGTGACCCCTTCC	GTGTTGATAGCGACGAGGTTGC
NO05G01620	GTCCTCCGCTGCCGCTTTC	CCAACGGGTCCCAGATGCC
NO25G00860	CCCGCCCCCAAGTTCTCC	GGGGGATGCCGTCACCTG
NO13G02860	CCCTGAGTTCGATCCCCTTGGCTTG	CAGGGCATCCACAGGCTTGGAC
NO16G01150	CTGCCACAAACAACAAGATGAC	GTTGAAGAACTCCTCGTTGTCC
NO09G03110	GTGATTTCCACCTTGCTCCAGTC	CGTGGCGGAGAAGTTAGTATCG
NO20G01990	TTTCGACTTTACCGTGGACTCGGAC	CTTGCAGTACTCGACAGCCTTGTCG
α-tubulin	CCGTTTGCCTGCCAAGTCCC	CACACGGTCGGCTCCAAGTCAAC
Actin	GACGGCACCAAGGTCAAAAT	ACGACGTGGAAGAGGAGGAA
