# Supplementary figures and images for: Enhanced Eicosapentaenoic Acid Production via Synthetic Biological Strategy in Nannochloropsis oceanica
Source: Mar Drugs. 2024 Dec 19;22(12):570. doi: 10.3390/md22120570 (PMC11676929; doi:10.3390/md22120570)

a

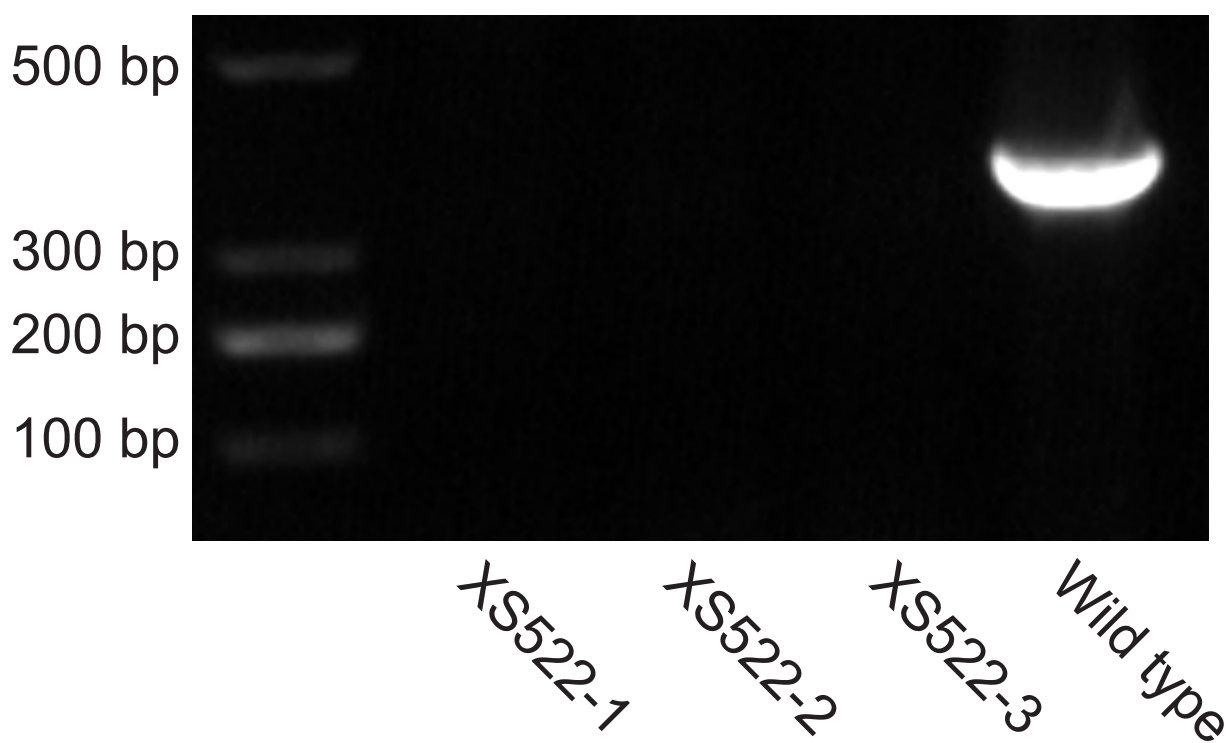

b

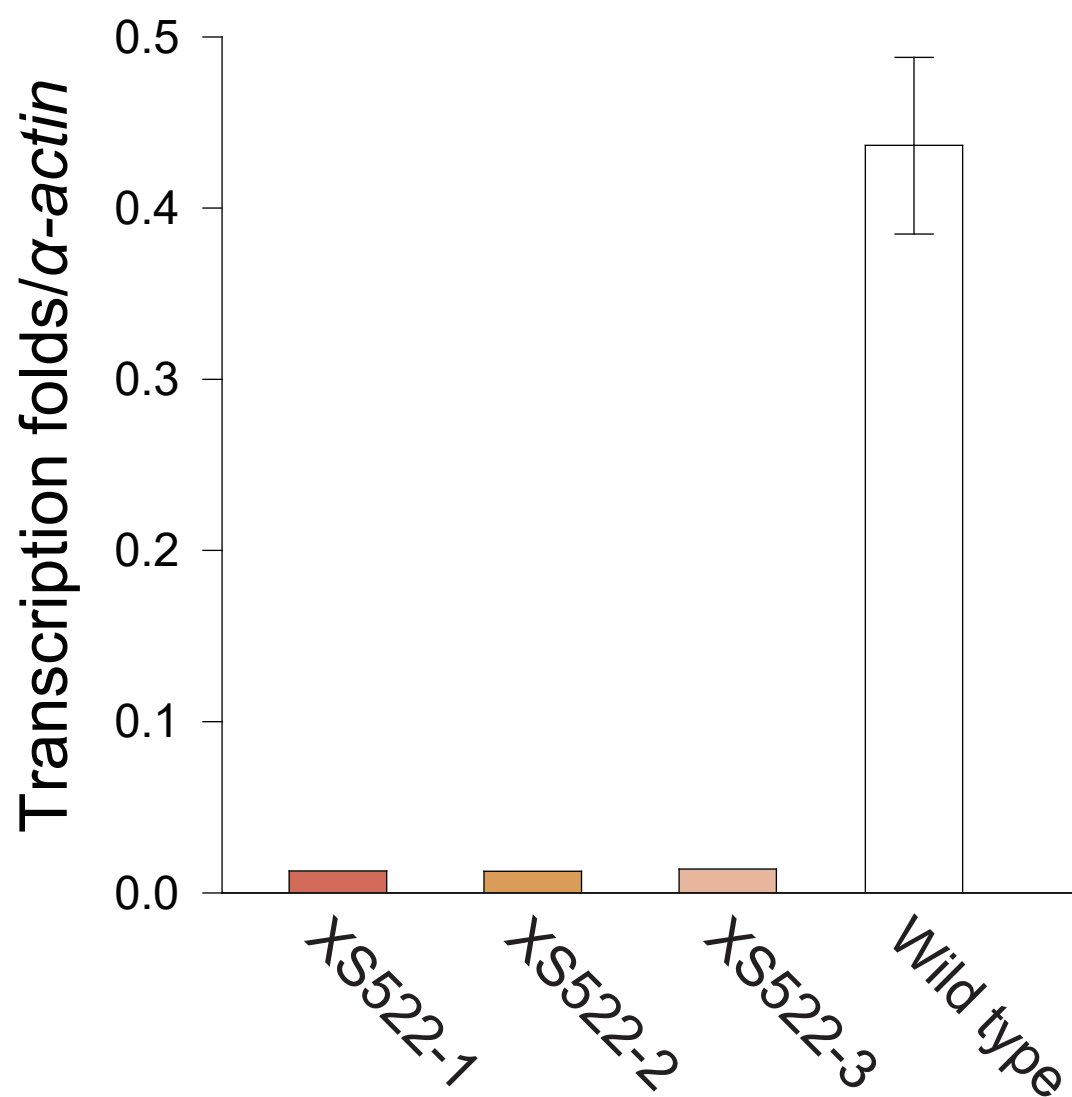

Supplement: Supplementary file 1 [file marinedrugs-22-00570-s001.zip › Supplementary Files/Figure S1.pdf]
